# Dietary Bioactive Compounds and Health

**DOI:** 10.3390/foods11162395

**Published:** 2022-08-10

**Authors:** Bonggi Lee, Choon Young Kim

**Affiliations:** 1 Department of Food Science and Nutrition, Pukyong National University, Nam-gu, Daeyeon-dong, Busan 608737, Korea; 2 Department of Food and Nutrition, Yeungnam University, Gyeongsan 38541, Korea

Foods primarily obtained from plant materials, such as fruits, vegetable, grains, legumes and other plant foods, provide not only nutrients but also non-nutrients. In addition to nutrients, non-nutrients in plant foods are bioactive compounds such as phytochemicals (polyphenols, terpenoids, alkaloids, phytosterols, and organosulfur compounds) and dietary fiber. A bioactive compound is generally defined as a substance with the biological potential to influence health in a beneficial way.

Accumulating studies support that dietary bioactive compounds and/or whole plant extracts have been linked to health promotion and disease prevention. These natural bioactive compounds have been known to have various biological functions including antioxidant, anti-inflammatory, anti-obesity, and anti-cancer activities. Thus, dietary bioactive compounds show preventive and/or therapeutic effects for various diseases such as obesity, diabetes, cancer, and skin diseases. Even though pure isolated bioactive compounds have potent biological actions, it is also important to investigate the benefits of plant extracts for human health. As a result of the combined action of multiple compounds within them, plant extracts exhibit additive, antagonistic or synergistic activity.

This Special Issue aims to provide scientific evidence focusing on all aspects of bioactive compounds with health benefits. This Special Issue includes several studies about the various biological effects of natural products and their derived compounds on multiple diseases related to metabolic syndrome, cachexia, melanogenesis, tumors, and bone loss. The Special Issues also covers two review papers including nutrients against glucocorticoid-induced muscle atrophy [1] and the health-beneficial properties of oats [2].

(1) Obesity and related metabolic abnormalities are closely related to multiple metabolic diseases and cancers. Marzo et al. examined the beneficial effects of apple extract on neoplastic lesions and cachexia using Crl:CD-1 mice treated with azoxymethane (AOM) and a high-fat diet to induce colon polyps related to obesity. After the treatment with AOM, Crl:CD-1 mice were fed either a high-fat diet or a high-fat diet supplemented with apple extract at 1% or 1.5%. The treatment with apple extract did not affect body weight, energy expenditure, or respiratory quotient but ameliorated signs of cachexia. The apple-extract-treated group exhibited lower sucrase, dipeptidyl-peptidase IV, and aminopeptidase N activities, and fewer intestinal lesions [3].
(2) Hwang et al. tested the cholesterol-lowering effect of the ethanol extract of capsella bursa-pastoris (CBE) using diet-induced obese mice and HepG2 cells [4]. The oral supplementation of CBE notably decreased the obesity-induced increase in blood LDL cholesterol concentration, which is closely related to the hepatic down-regulation of pro-protein convertase subtilisin/kexin type 9 (PCSK9), a gene related to lysosomal degradation of LDL receptor. When HepG2 cells were cultured in delipidated serum, the intracellular protein levels of PCSK9 were significantly increased, but CBE treatment decreased it without altering LDL receptor levels. Further analysis revealed that icaritin contained in CBE may be one of the functional compounds to explain those effects. These data indicate that CBE and icaritin can be applied for the treatment of hypercholesterolemia.
(3) Azam et al. tested the anti-melanogenic activity of sargahydroquinoic acid (SHQA) abundant in the brown alga *Sargassum serratifolium* using B16F10 cells treated with alpha-melanocyte-stimulating hormone (α-MSH). The treatment of SHQA significantly reduced cellular tyrosinase activity and melanin content partly by downregulating the expression of tyrosinase and tyrosinase-related protein 1. Further experiments revealed that SHQA inhibited the main transcription factors for melanogenesis, including microphthalmia-associated transcription factor and cAMP-responsive element-binding protein, indicating that SHQA may be applied to a pharmaceutical or cosmetic agent against hyperpigmentation [5].
(4) Although various biological activities, including the antioxidant and anti-tumor effects of the extract of *Rubus coreanus* Miquel (*R. coreanus*), have been reported, the effects on the PD-1/PD-L1 immune checkpoint have not been tested. Kim et al. proved that the water extract of *R. coreanus* suppressed the binding of PD-1 to PDL1 using competitive enzyme-linked immunosorbent assay (ELISA) and cell-based bioassay [6]. When its anti-tumor effect was tested in vivo using humanized PD-1 mice bearing MC38 colorectal tumors, the oral administration of the water extract exhibited anti-tumor activity similar to anti-PD-1 antibody, at least partly by dose-dependent blocking of PD-1 binding to PD-L1. Additionally, further study revealed that ellagic acid is the main compound underlying the anti-tumor and immune-checkpoint-regulatory effects of *R. coreanus* extract [6].
(5) Jang et al. examined the effects of the water extract of *Agastache rugosa*, an aromatic herb widely used as a food ingredient, on bone health. They focused on the beneficial effects of *A. rugosa* on osteoclast differentiation and bone loss in ovariectomized mice that can be used as a model for postmenopausal osteoporosis [7]. The oral supplementation of the water extract notably ameliorated ovariectomy-mediated trabecular bone loss and fat accumulation in the bone marrow, in which the effects are related to the inhibition of receptor activator of nuclear factor-κB ligand (RANKL)-induced osteoclast differentiation in osteoclast precursor cells and anti-resorption activity on a bone mimetic surface [7]. Further analysis identified seventeen phytochemicals, including five phenols and twelve flavonoids, of which eleven compounds exhibited anti-osteoclastogenic effects, indicating that the water extract of *A. rugosa* may be used as a therapeutic agent against postmenopausal osteoporosis [7].
